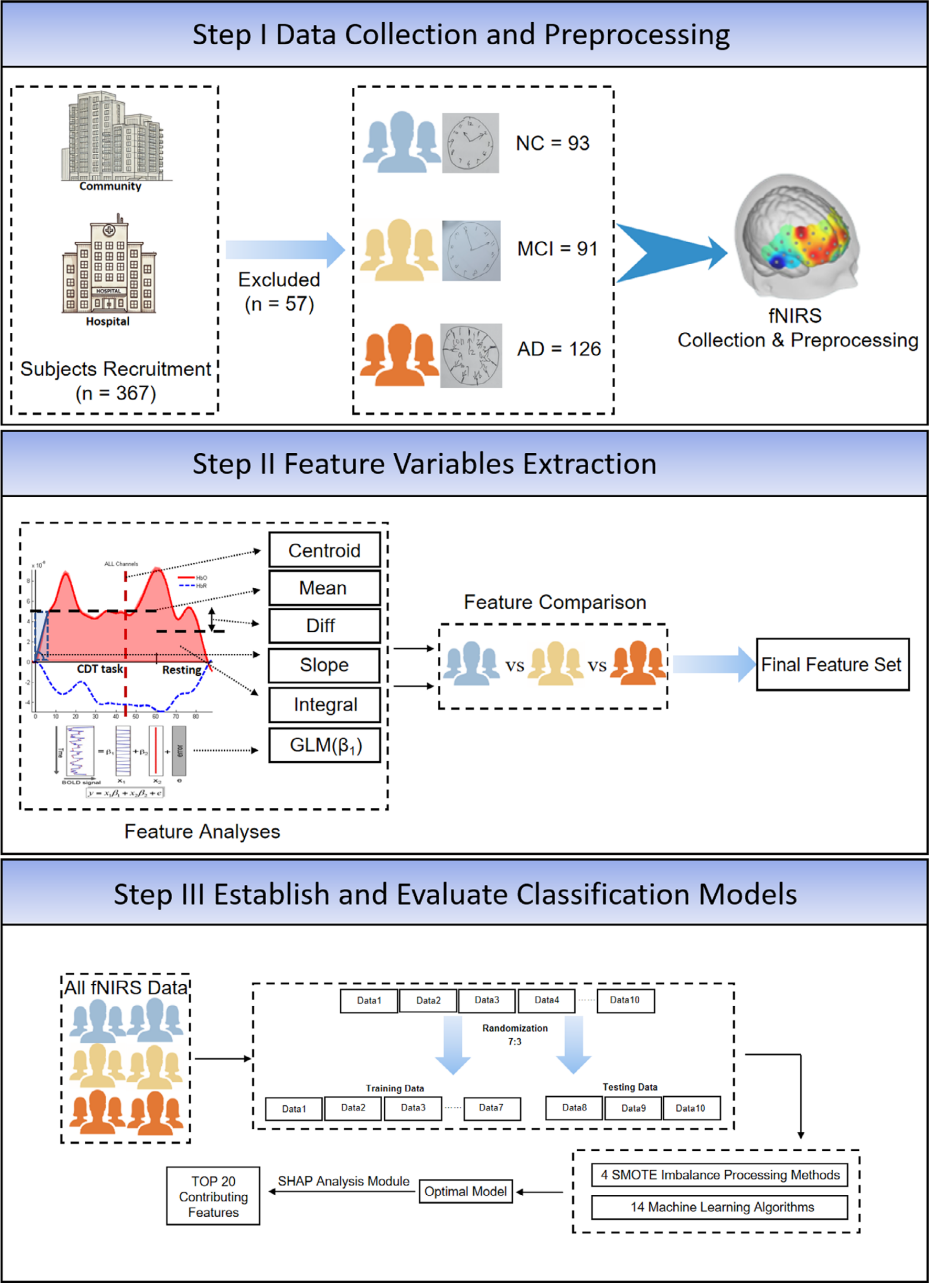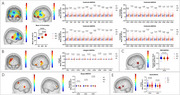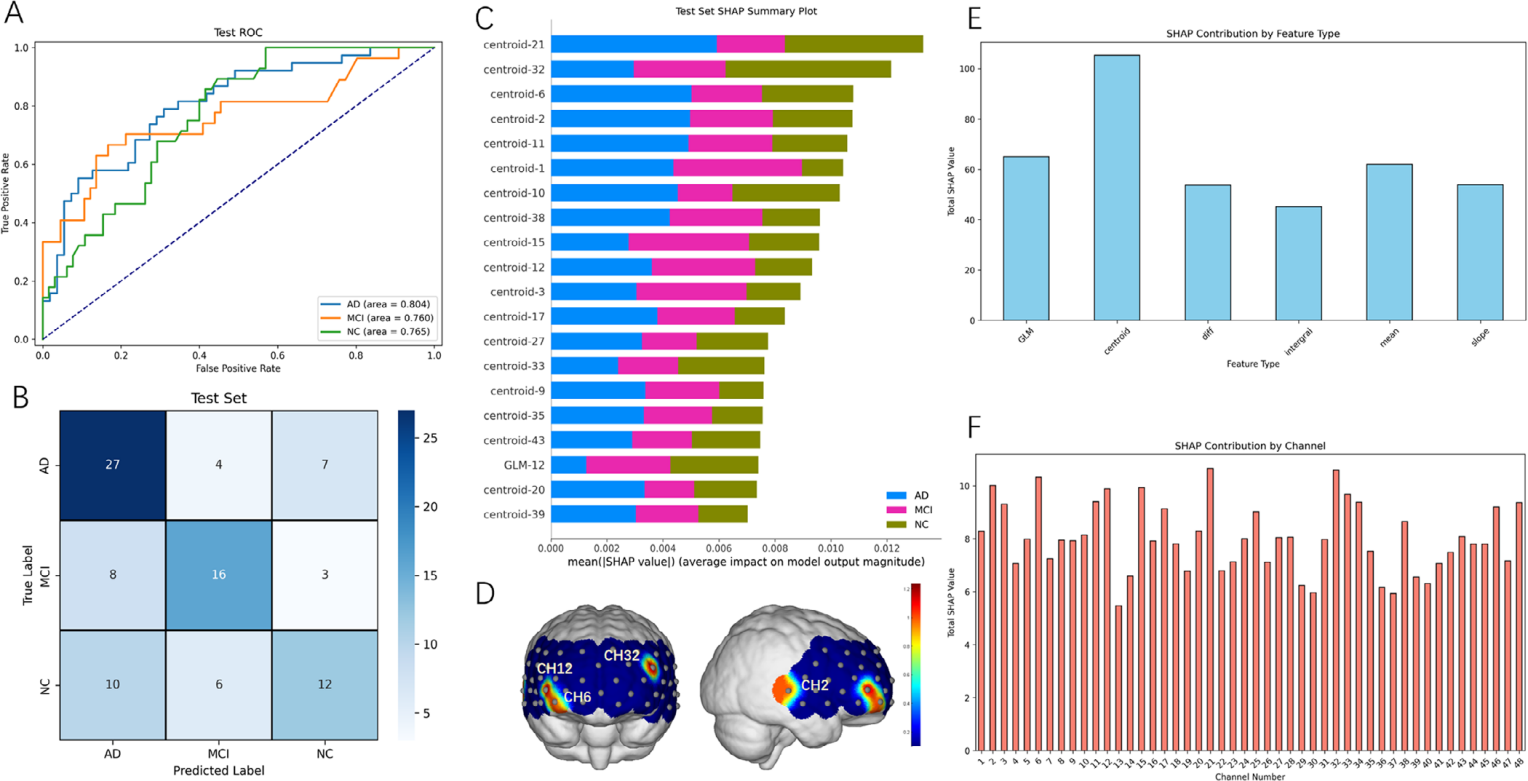# Early Detection of Cognitive Impairment Using fNIRS and Machine Learning: Insights from the Clock Drawing Test

**DOI:** 10.1002/alz70856_101852

**Published:** 2025-12-25

**Authors:** Wenbo Zhang, Ming Chen, Fuxin Zhong, Zekung Jiang, Kang Li, Weihua Yu, Yang Lü

**Affiliations:** ^1^ The First Affiliated Hospital of Chongqing Medical University, Chongqing, Chongqing, China; ^2^ West China Hospital of Sichuan University, Chengdu, Sichuan, China; ^3^ Chongqing Medical University, Chongqing, Chongqing, China

## Abstract

**Background:**

This study aimed to address the challenge of early detection and differentiation of cognitive impairment stages, including normal cognition (NC), mild cognitive impairment (MCI), and Alzheimer's disease (AD), by utilizing functional near‐infrared spectroscopy (fNIRS) during the Clock Drawing Test (CDT).

**Method:**

Neural activation during CDT was recorded using a multi‐channel fNIRS system, was analyzed using the generalized linear model (GLM), in addition with features as Integral, Centroid, Slope, Difference (Diff), and Mean were extracted from Oxy‐Hb signals. Based on the fNIRS features, we further developed and validated a cognitive progression prediction model using machine learning pipeline for three‐class classification. Shapley Additive Explanations (SHAP) analysis was used to interpret feature importance and localize critical brain regions.

**Result:**

A total of 310 participants (93 NC, 91 MCI, 126 AD) aged 60–90 years were recruited. The centroid values across all 48 channels showed significant differences among NC, MCI, and AD groups (*P* < 0.05, FDR corrected). The overall mean centroid value increased progressively from NC (32.98 ± 1.72s) to AD (48.56 ± 2.68s), with discontinuous changes in specific channels where MCI exhibited the smallest values. The Diff value (task‐rest change in Oxy‐Hb) was notably lower in MCI for CH16 (left superior temporal gyrus, *P* = 0.037), while the MCI group showed the steepest initial Oxy‐Hb increase across multiple channels (*P* < 0.05). GLM‐derived β values revealed the greatest activation in NC and the smallest in MCI for channels overlapping Broca's area and the superior temporal gyrus. Out of 65 machine learning model and combined with 5 unbalanced processing techniques, Synthetic Minority Over‐sampling Technique (SMOTE) and the Extra Trees achieved a macro‐average AUC of 0.776, with SHAP analysis identifying features from the dorsolateral prefrontal cortex, superior temporal gyrus, and Broca's area as critical for classification.

**Conclusion:**

Combining fNIRS and machine learning enhances early detection of cognitive impairment, particularly MCI, with improved accuracy and clinical insights into neural mechanisms underlying cognitive decline.